# Development of a novel endolysin, PanLys.1, for the specific inhibition of *Peptostreptococcus anaerobius*

**DOI:** 10.5713/ab.22.0454

**Published:** 2023-05-02

**Authors:** Joonbeom Moon, Hanbeen Kim, Dongseok Lee, Jakyeom Seo

**Affiliations:** 1 Department of Animal Science, Life and Industry Convergence Research Institute, College of Natural Resources and Life Sciences, Pusan National University, Miryang 50463, Korea

**Keywords:** Endolysin, Hyper-ammonia-producing Bacteria, *Peptostreptococcus anaerobius*

## Abstract

**Objective:**

The objective of this study was to develop a novel endolysin (PanLys.1) for the specific killing of the ruminal hyper-ammonia-producing bacterium *Peptostreptococcus anaerobius* (*P. anaerobius*).

**Methods:**

Whole genome sequences of *P. anaerobius* strains and related bacteriophages were collected from the National Center for Biotechnology Information database, and the candidate gene for PanLys.1 was isolated based on amino acid sequences and conserved domain database (CDD) analysis. The gene was overexpressed using a pET system in *Escherichia coli* BL21 (DE3). The lytic activity of PanLys.1 was evaluated under various conditions (dosage, pH, temperature, NaCl, and metal ions) to determine the optimal lytic activity conditions. Finally, the killing activity of PanLys.1 against *P. anaerobius* was confirmed using an *in vitro* rumen fermentation system.

**Results:**

CDD analysis showed that PanLys.1 has a modular design with a catalytic domain, amidase-2, at the N-terminal, and a cell wall binding domain, from the CW-7 superfamily, at the C-terminal. The lytic activity of PanLys.1 against *P. anaerobius* was the highest at pH 8.0 (p<0.05) and was maintained at 37°C to 45°C, and 0 to 250 mM NaCl. The activity of PanLys.1 significantly decreased (p<0.05) after Mn^2+^ or Zn^2+^ treatment. The relative abundance of *P. anaerobius* did not decrease after administration PanLys.1 under *in vitro* rumen conditions.

**Conclusion:**

The application of PanLys.1 to modulate *P. anaerobius* in the rumen might not be feasible because its lytic activity was not observed in *in vitro* rumen system.

## INTRODUCTION

Feed protein ingested in the rumen is degraded by rumen proteolytic bacteria and then converted to amino acids, which are subsequently degraded into ammonia and residual carbon skeletons. Although ammonia is an essential nitrogen source for rumen microbial growth, its production by excessive feed protein degradation in the rumen is associated with inefficient nitrogen utilization in ruminants [1]. When the amount of feed protein in the rumen exceeds the amount required by rumen microorganisms, ammonia is produced and metabolized to urea, resulting in the dissipation of nitrogen in the urine [2].

Hyper-ammonia-producing bacteria (HAB), identified as *Clostridium aminophilum*, *C. sticklandii*, and *Peptostreptococcus anaerobius*, are gram-positive and monensin-sensitive bacteria that grow in the rumen using amino acids as their sole carbon, nitrogen, and energy source [3,4]. They are known to generate ammonia faster than other proteolytic bacteria [5]. Thus, there have been several studies on improving nitrogen utilization by inhibiting HAB using several antimicrobial additives, such as ionophores [6–8] and plant extracts [9,10]. Among the HAB, *P. anaerobius* produces ammonia mainly by utilizing leucine, serine, phenylalanine, threonine, and glutamine at faster rates, and their ammonia yield is nearly 3-fold higher than those of *C. aminophilum* and *C. sticklandii* [3]. Moreover, in a previous *in vitro* enrichment culture study, Shen et al [11] reported that the increase in ammonia concentration was due to HAB, and *Peptostreptococcus* (*P. anaerobius*) was positively correlated with ammonia production (up to 41%, p<0.01).

Endolysins produced from bacteriophages are hydrolases that can degrade the bacterial peptidoglycan (PG) layer to release progeny phages at the end of the bacteriophage replication cycle [12]. Endolysins targeting gram-positive bacteria have a modular structure and consist of two distinct domains: the cell wall-binding domain (CBD) at the C-terminal and an enzymatically active domain (EAD) at the N-terminal [13]. Because of the CBD, endolysins exhibit host-specific bactericidal activity without killing the beneficial microbiota [12]. In addition, many studies have shown that exogenous application of recombinant endolysin can also rapidly kill target bacteria with a low risk of resistance [12]. For this reason, endolysins are promising candidates for replacing antibiotics in various fields to modulate specific bacteria. The traditional strategy to develop recombinant endolysins is to screen appropriate bacteriophages to kill target bacteria and acquire gene information with respect to endolysins from selected bacteriophages [14,15]. Recently, however, several studies have shown that putative endolysin genes identified by bioinformatics tools can be used to develop recombinant endolysins without laborious work to find bacteriophages [16,17]. In a previous study, the endolysin LyJH307 targeting ruminal bacteria, *Streptococcus bovis*, was developed using gene information extracted by bioinformatics tools and successfully showed an inhibitory effect against target bacteria [17].

In the present study, we hypothesized that a recombinant endolysin which can recognize *P. anaerobius* can specifically hydrolyze the target bacterium. Thus, the objective of this study was to develop a novel endolysin with lytic activity against *P. anaerobius* using genomic information on the *P. anaerobius* specific phage and to evaluate its optimal lytic conditions.

## MATERIALS AND METHODS

### Bacterial strains and growth conditions

*Escherichia coli* cloning strain DH5*α* and expression strain BL21 (DE3) were aerobically grown at 37°C in Luria-Bertani (LB) broth (Difco Laboratories Inc., Detroit, MI, USA). *P. anaerobius* (KCTC 5182) was purchased from the Korean Collection for Type Cultures (Jeongeup, Korea) and was used to identify the lytic activity of the recombinant endolysin developed in this study. *P. anaerobius* was anaerobically grown at 39°C in a basal medium containing (per L): 292 mg KH_2_PO_4_, 292 mg K_2_HPO_4_, 480 mg (NH_4_)_2_SO_4_, 480 mg NaCl, 268 mg NH_4_Cl_2_, 100 mg MgSO_4_·7H_2_O, 64 mg CaCl_2_·2H_2_O, 600 mg cysteine hydrochloride, 4 g Na_2_CO_3_, 15 g casamino acids (Difco Laboratories Inc., USA), and 15 g yeast extract (Difco Laboratories Inc., USA) adjusted to pH 6.8.

### Identification, cloning, and overexpression of the endolysin PanLys.1

Whole genome sequences of *P. anaerobius* strains and relatives were retrieved from the National Center for Biotechnology Information (NCBI) genome browser and annotated using PhiSpy [18] to identify putative endolysin genes. Based on the lysis module analysis, one putative endolysin gene was selected and named PanLys.1. The putative gene for PanLys.1 was synthesized as an *E. coli* codon-optimized construct and cloned into the expression vector pET28b (Novagen Inc., Madison, WI, USA) containing an N-terminal hexa-histidine tag (6×His tag) sequence by Bionics Inc. (Seoul, Korea). The cloned plasmid was transformed into competent *E. coli* BL21 (DE3) cells that were grown in LB broth until the optical density at 600 nm (OD_600 nm_) reached 0.4. Thereafter, 1 mM isopropyl-β-D-thiogalactoside was added to the medium, and the cells were further incubated for 18 h at 16°C. Harvested cells were suspended in lysis buffer (50 mM NaH_2_PO_4_, 300 mM NaCl, 10 mM imidazole, pH 8.0) and lysed by sonication (KYY-80; Korea Process Technology Co., Ltd., Seoul, Korea). After centrifugation at 10,000×g for 15 min, the supernatant was passed through Ni-NTA agarose (Qiagen GmbH, Hilden, Germany) and the recombinant PanLys.1, purified as described by the manufacturer, was resolved using sodium dodecyl sulfate-polyacrylamide gel electrophoresis (SDS-PAGE). The purified endolysin was pooled and dialyzed against the elution buffer (50 mM NaH_2_PO_4_, 300 mM NaCl, and pH 8.0).

### Amino acids alignment and structure prediction of PanLys.1

Based on the results of the conserved domain database (CDD) analysis, amino acid sequences corresponding to the EAD and CBD of PanLys.1 were collected according to Bustamante et al [19]. Multi-alignment based on amino acid sequences was carried out using the L-INS-I algorithm implemented in multiple alignment using fast fourier transform (MAFFT v7.505) [20], and the results of alignment was visualized using ggmsa in R software (version 4.2.1) [21]. The amino acid sequences of PanLys.1 were uploaded to ColabFold for the prediction of the three-dimensional structures of PanLys.1 [22]. The predicted structure with the highest predicted local distance difference test score was visualized using UCSF ChimeraX (version 1.3) [23].

### Characterization of PanLys.1

The lytic activity of PanLys.1 was assayed as a decrease in OD_600 nm_ [17]. To determine the dose-dependent response, *P. anaerobius* (KCTC 5182) was cultivated to an OD_600 nm_ of 0.8 to 1.0, harvested, and resuspended in the same amount of 50 mM sodium phosphate buffer (pH 8.0). Serially diluted endolysin (20 μL, 3.125 to 100 μg/mL) was added to 96-well cell plates (SPL Life Sciences Co., Ltd., Pocheon, Korea) along with cell suspensions (180 μL) and incubated at 39°C. The OD_600 nm_ values were monitored every 30 min for 120 min using an iMark microplate reader (Bio-Rad Laboratories Inc., Hercules, CA, USA). The optimal temperature was determined by measuring the lytic activity of PanLys.1 (100 μg/mL) at 4°C, 16°C, 25°C, 37°C, and 45°C. The optimal pH was determined by suspending *P. anaerobius* (KCTC 5182) in 50 mM sodium acetate buffer (pH 4.5 to 5.5) and 50 mM phosphate buffer (pH 6.0 to 8.0). The influence of NaCl concentration on PanLys.1 activity was tested by adding 31.25, 62.5, 125, 250, and 500 mM NaCl to the empirically determined optimal pH buffer. The effects of divalent cations were determined as previously described [17]. PanLys.1 (100 μg/mL) was incubated with 5 mM ethylenediaminetetraacetic acid (EDTA) at 25°C for 30 min to chelate divalent cations attached to the endolysin. EDTA was removed by replacing the buffer with an empirically determined buffer at the optimal pH using an Amicon Ultra-4 (10 kDa) (Merck KGaA, Darmstadt, Germany). The lytic activities of the endolysins incubated with EDTA, 10 mM CaCl_2_, MgCl_2_, MnCl_2_, and ZnCl_2_ were assessed. All experiments were conducted in triplicate.

### *In vitro* rumen fermentation

Three experimental treatments were used as follows: i) Control, 10 mL of *in vitro* buffer with 1% *P. anaerobius* (100 μL); ii) Positive control, control with 1 mL of elution buffer; iii) Endolysin, control with 1 mL of PanLys.1. The *in vitro* fermentation was approved by the Animal Research Ethics Committee of Pusan National University (Pusan, Korea, PNU-2020-2827) and was performed using rumen fluid collected from two cannulated Holstein steers (body weight = 450±30 kg) before the morning feeding at the Center for Agriculture Research, Pusan National University, Korea. The steer was fed a diet of 600 g/kg Timothy hay and 400 g/kg of a commercial concentrate mix (Farmsco Co., Ltd., Anseong, Korea). Rumen fluid was collected before the morning feeding time, mixed, transferred into a thermos bottle, and immediately transported to the laboratory. The rumen contents were filtered through two layers of cheesecloth and mixed with 4× volume of *in vitro* rumen buffer solution [24]. Four bottles were used per treatment, and 10 mL of the rumen fluid and buffer mixture was transferred, accompanied by continuous flushing with O_2_-free CO_2_ gas. The bottles were sealed with butyl rubber stoppers and aluminum caps and incubated on a rotary shaker (JSSI-300T; JS Research Inc., Gongju, Korea) at 20 rpm for 6 h at 39°C. After 6 h of incubation, 1 mL of the elution buffer (without endolysin) and PanLys.1 were injected into the respective bottles (Positive control and Endolysin) using sterilized syringes, and the bottles were incubated for 2 h. After 2 h of incubation, the bottle caps were removed, and the bottles were fixed immediately on ice to stop fermentation. The sample fluid (2 mL) was centrifuged at 20,000×g for 20 min at 4°C, and then the supernatant was discarded, and the pellet was stored at −80°C until rumen microbial population analysis.

### DNA extraction and real-time polymerase chain reaction

Total DNA was extracted from the pellet stored at −80°C using the repeated bead beating plus column (RBB+C) method [25]. Genomic DNA was treated with RNase A and proteinase K and purified using columns from the DokDo-Prep Genomic DNA Kit (Elpis-Biotech, Daejeon, Korea). The total DNA concentration and purity were measured using a Nanodrop spectrophotometer (ND-1000; Thermo Fisher, Waltham, MA, USA). The purified DNA was stored at −20°C until amplicon sequencing and real-time polymerase chain reaction (PCR). To assess the relative abundance of rumen microbes among the treatments, real-time PCR assays were performed on a CFX 96 Touch system (Bio-Rad Laboratories Inc., USA) using general bacterial primer sets (forward, CGGCAAC GAGCGCAACCC; reverse, CCATTGTAGCACGTGTG TAGCC; efficiency, 1.898) [26] and *P. anaerobius* primer sets (forward, GCATTCAGTTGGGCACTCTA; reverse, CGT GTGTAGCCCTAAGCATAA; efficiency, 1.899, in this study). Primer efficiency was calculated as follows:


$$\text{Efficiency}=10^{-1/\text{slope}}$$

Reactions were performed in triplicate, in reaction volumes of 20 μL, using optical reaction plates sealed with an optical adhesive film. Each reaction mixture contained 0.5 μL of 10 mM dNTP Mix (BioFACT, Daejeon, Korea), 2 μL of 10× buffer (BioFACT, Korea), 1 μL of genomic DNA diluted 10-fold, 1 μL of forward primer (10 μM), 1 μL of reverse primer (10 μM), 0.1 μL of Taq polymerase (BioFACT, Korea), 1 μL of Evagreen (SolGent Co., Ltd., Daejeon, Korea), and 13.4 μL of PCR-grade water. Real-time PCR was carried out according to the manufacturer’s instructions, as follows: initiation for one cycle at 95°C for 10 min; 40 cycles of denaturation at 95°C for 30 s, annealing at 60°C for 30 s, and elongation at 72°C for 30 s; and final elongation at 72°C for 5 min. Fluorescence was recorded at the end of each denaturation and extension step, and the specificity of the amplicon was confirmed via dissociation curve analysis of PCR end-products by increasing the temperature from 60°C to 95°C at a rate of 1°C every 30 s. ‘General bacteria’ was used as the reference gene. A comparative threshold cycle method was used to calculate the fold change of *P. anaerobius* compared with that in the control [27].

### Statistical analysis

Statistical analysis was conducted using R software (R version 4.1.1; R Foundation for Statistical Computing, Vienna, Austria) to compare differences in the lytic activity of PanLys.1 under different conditions. A non-parametric Kruskal–Wallis test using the function kruskal.test was applied because the residuals did not follow a normal distribution. Differences among groups were compared using the Dunn’s multiple comparison test using the dunnTest function from the FSA package, if a significant effect was observed. All p-values were adjusted using the Benjamini-Hochberg false discovery rate. Statistical analysis of the relative abundance of *P. anaerobius* in the *in vitro* experiment was performed using the PROC GLIMMIX procedure in SAS 9.3 (SAS Institute Inc., Cary, NC, USA). Differences among treatments were compared using Tukey’s range test, if a significant effect was observed. Statistical significance was set at p<0.05.

## RESULTS AND DISCUSSION

### Sequence analysis and overexpression of PanLys.1

**Figure 1:**
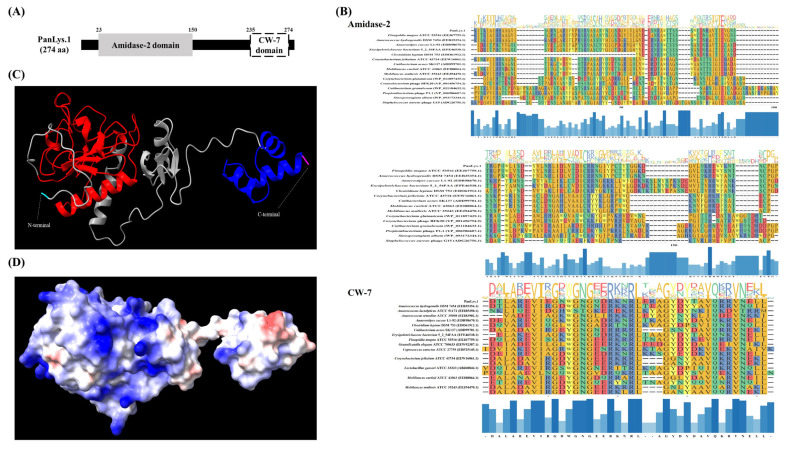
Domain and structure analysis of endolysin PanLys.1. (A) Conserved domain of PanLys.1. The gray square represents the N-terminal enzymatically active domain (amidase-2), and the white square represents the C-terminal cell wall binding domain (CW-7 domain); (B) Sequence conservation in the amidase-2 and CW-7 domains. The domains were multi-aligned based on amino acid sequence using an L-INS-I multiple alignment algorithm on multiple alignment using fast fourier transform (version 7.505) and results were visualized using the ggmsa package in R (version 4.2.1); (C) Three-dimensional model of PanLys.1 predicted by AlphaFold2 on ColabFold notebook which had the highest predicted local distance difference test score. The ribbon form shows the amidase-2 (red) and CW-7 (blue) domains of PanLys.1; (D) Electrostatic potential of PanLys.1 created with ChimeraX (version 1.3). The positive and negative potentials are shown in blue and red, respectively.

**Figure 2:**
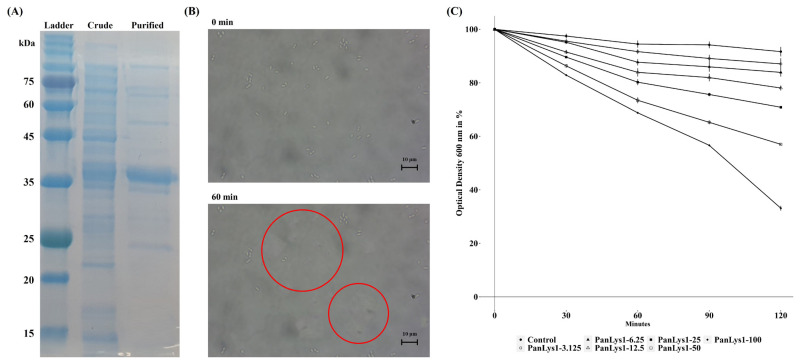
Purification of PanLys.1 and its lytic activity against *Peptostreptococcus anaerobius* (KCTC 5182). (A) Purified PanLys.1 resolution on 15% SDS-PAGE. Lane 1, stained protein molecular weight markers; Lane 2, crude form of PanLys.1; Lane 3, purified form of PanLys.1; (B) Optical microscopic image of the lytic activity of PanLys.1 against *P. anaerobius*. *P. anaerobius* was cultured overnight, harvested, and suspended in 50 mM sodium phosphate buffer (pH 8.0). Suspensions of *P. anaerobius* (45 μL) were mixed with PanLys.1 after incubation with 10 mM MgCl_2_ (5 μL, 100 μg/mL) and visualized at 1,000× magnification. Live cells of *P. anaerobius* are shown in red circles, and the disappearance of white cells in the circle indicates the death of the cell; (C) *P. anaerobius* was incubated with various doses of PanLys.1 or elution buffer. Data are presented as means±standard deviation of triplicates. SDS-PAGE, sodium dodecyl sulfate-polyacrylamide gel electrophoresis; OD, optical density.

Amino acid sequence analysis using the NCBI conserved domain showed that PanLys.1 had a modular design with two distinct domains: at the N-terminal, amidase-2, a member of the peptidoglycan recognition protein (PGRP) superfamily (cl02712, *e*-value = 5.68×10^−16^) with enzymatic activity, and at the C-terminal, CW-7 of the CW-7 superfamily (cl07020, *e*-value = 3.03×10^−19^), which attaches to the PG layer of the bacterial cell wall, similar to most endolysins from bacteriophages that infect gram-positive bacteria [12] (Figure 1A). Amidase-2 is an N-acetylmuramoyl-L-alanine amidase that hydrolyzes the lactyl-amide bond between N-acetylmuramic acid and L-alanine, the first amino acid in the stem peptide [28]. In general, amidase-2 consists of five central β-sheet strands, mostly parallel, and several peripheral α-helices. The enzyme has a conserved Zn^2+^-binding site in the PG-binding groove and consists of two histidines and a cysteine residue, which are highly conserved and are essential for enzymatic activity [28]. The amidase-2 of PanLys.1 was also has conserved amino acid regions and the same fold, consisting of five parallel strands and helices (Figure 1C). The putative CBD at the C-terminal of PanLys.1 is a member of the CW-7 superfamily. This domain is found at the C-terminal region of the Cpl-7 lysozyme, encoded by the pneumococcal bacteriophage Cp-7, and contains a variable number (1 to 3) of repeated motifs (39 to 42 amino acid-long segments) [19]. These CW-7 repeats are composed of three-helix bundle folds and are known to recognize and bind to N-acetyl-D-glucosaminyl-N-acetylmuramyl-L-alanyl-D-isoglutamine within the PG layer of the target bacteria [29]. The binding of CW-7 is mediated by its conserved residues (glycine and arginine with hydrophobic amino acids) that stabilize the enzyme through hydrophobic and polar interactions and create shallow grooves that contain a muropeptide-binding site [29]. The CW-7 domain of PanLys.1 was also has conserved amino acid regions and a predicted three-helix bundle fold structure (Figure 1C). Since PanLys.1 has a conventional structure (modular design, both catalytic and binding domains), a turbidity reduction assay was conducted to determine the lytic activity of PanLys.1 against *P. anaerobius*. Recombinant PanLys.1 was expressed in *E. coli* BL21 (DE3) and purified by nickel affinity chromatography using an N-terminal 6× His tag. The major band of the purified soluble PanLys.1 endolysin was resolved by SDS-PAGE at a molecular mass of 30.24 kDa (Figure 2A).

### Characterization of endolysin PanLys.1

**Figure 3:**
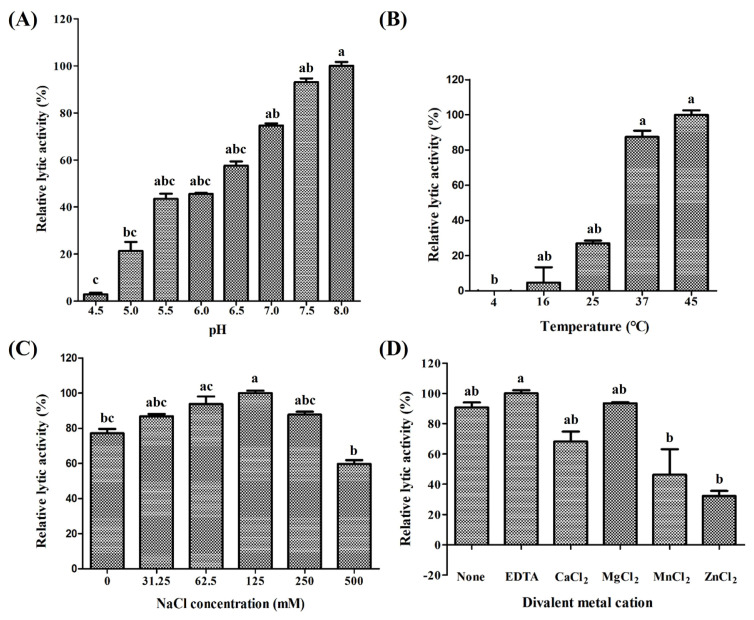
Identification of optimal conditions for the lytic activity of PanLys.1. *Peptostreptococcus anaerobius* (KCTC 5182) was incubated with 100 μg of PanLys.1 at various (A) pH, (B) temperatures, (C) NaCl concentrations, and (D) metal ions. Data are shown as means±standard deviation of triplicate samples. ^a–c^ Means with different letters differ (p<0.05).

*P. anaerobius* (KCTC 5182) was selected as the reference strain to measure the lytic activity of PanLys.1. PanLys.1 reduced the optical density of *P. anaerobius* dose-dependently above a concentration of 3.125 μg/mL, and at a concentration of 100 μg/mL, inhibited nearly 70% of *P. anaerobius* after 2 h of incubation (Figure 2B). Thus, the optimal lytic activity of PanLys.1 was measured at 100 μg/mL with a 2 h incubation time. Lytic activity was the highest at pH 8.0 (high between 6.5 and 8.0) (Figure 3A) and 45°C (maintained between 37°C and 45°C) (Figure 3B). Lytic activity of PanLys.1 was highest at 125 mM NaCl but was not affected by 0 to 250 mM NaCl (Figure 3C). Incubation with EDTA and Mg^2+^ did not influence lytic activity (increased by ~10%). However, Mn^2+^ and Zn^2+^ reduced the activity of PanLys.1 by ~55% and 70%, respectively (Figure 3D). The enzyme activity of amidase-2 is zinc-dependent, but the activity of PanLys.1 with 10 mM of ZnCl_2_ in this study decreased significantly. Based on the above observations, we hypothesized that PanLys.1 might have potent lytic activity against *P. anaerobius* under *in vitro* rumen conditions.

### *In vitro* rumen fermentation

**Figure 4:**
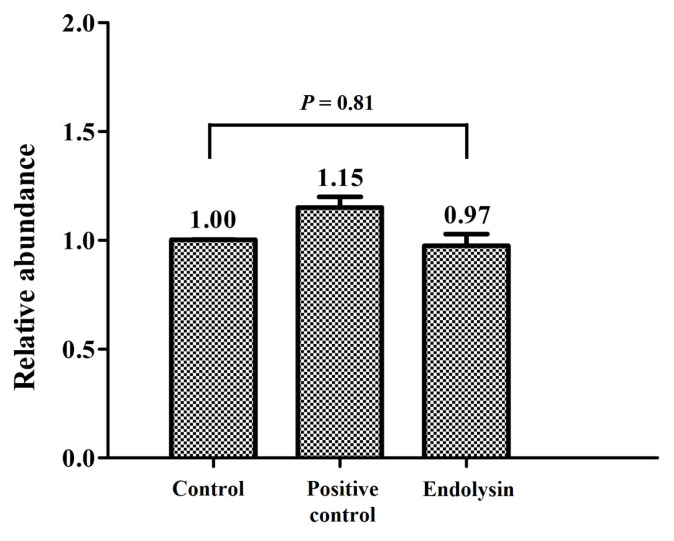
Relative abundance of *Peptostreptococcus anaerobius* after *in vitro* incubation. Control, no addition; Positive control, addition of 1 mL of recombinant protein elution buffer without endolysin; Endolysin, addition of 1 mL of elution buffer containing PanLys.1. Data are presented as means±standard deviation of four replicates.

Based on the characterization results, we assessed PanLys.1 activity against *P. anaerobius* during *in vitro* rumen fermentation, and the results of lytic activity are presented in Figure 4. The addition of PanLys.1 did not decrease the relative abundance of *P. anaerobius* during *in vitro* rumen fermentation (Figure 4; p = 0.81). It is assumed that rumen fluid, which contains high levels of cations, may affect the lytic activity of PanLys.1, because the lytic activity of PanLys.1 decreased when Mn^2+^ and Zn^2+^ were attached (Figure 3). It has been reported that various metal ions originating from mixed feedstuff, including Mn^2+^ and Zn^2+^, are observed in rumen fluid [30]. In addition, *in vitro* rumen fermentation buffer mixed with rumen fluid contains micro minerals for the proliferation of ruminal microorganisms. Therefore, in *in vitro* rumen fermentation, PanLys.1 might randomly attach to various metal ions, thereby compromising its lytic activity against *P. anaerobius*.

Similar to our study, several studies have reported that recombinant endolysin shows lytic activity only under well-controlled conditions, but not *in vivo*. Peptide-modified endolysin PlyA showed broad lytic activity against *Acinetobacter baumannii* and *Pseudomonas aeruginosa* in Tris-HCl buffer, but its antibacterial activity was not observed in LB culture medium, pasteurized milk, and serum [31]. Vouillamoz et al [32] evaluated the antibacterial activity of Cpl-1, an endolysin of bacteriophage Cp-1, in a mouse model of pneumococcal bacteremia. Mice infected with *Streptococcus pneumoniae* strain D39 were treated with Cpl-1, but the mice did not survive over 72 h, even though Cpl-1 showed high lytic activity in sodium phosphate buffer in a previous study [33]. Oliveira et al [34] suggested that the differences in lytic activity in buffered solutions and *in vivo* environments resulted from the state of target bacteria due to the presence or absence of nutrients in the tested solution. Target bacteria enter a lag phase temporarily, resulting in their PG layers becoming physiologically unstable and susceptible to the enzymes in the buffer-suspended lytic activity test. However, bacteria in high-nutrient conditions are metabolically active, and their PG structure becomes more complicated because of the acetylation of glycan chains and amidation of peptides, leading to resistance to enzymes [34]. In conclusion, the difference in lytic activity in the buffer-suspended test and *in vitro* rumen fermentation might be a result of the inability of PanLys.1 to act on growing *P. anaerobius* due to the maturity of the PG layer in high-nutrient environments.

## CONCLUSION

In the present study, we developed a novel endolysin, PanLys.1, using a genomic database, and then confirmed its lytic activity against *P. anaerobius*. PanLys.1 has a consecutive modular structure composed of amidase-2 and CW-7 domains. PanLys.1 had an optimal lytic activity at pH 6.5 to 8.0 and a ruminal temperature of 39°C. However, lytic activity was not observed in an *in vitro* rumen system; therefore, the application of PanLys.1 to kill *P. anaerobius* in the rumen might not be feasible. Further studies are needed to improve and verify the effect of PanLys.1 on rumen microbiota.
